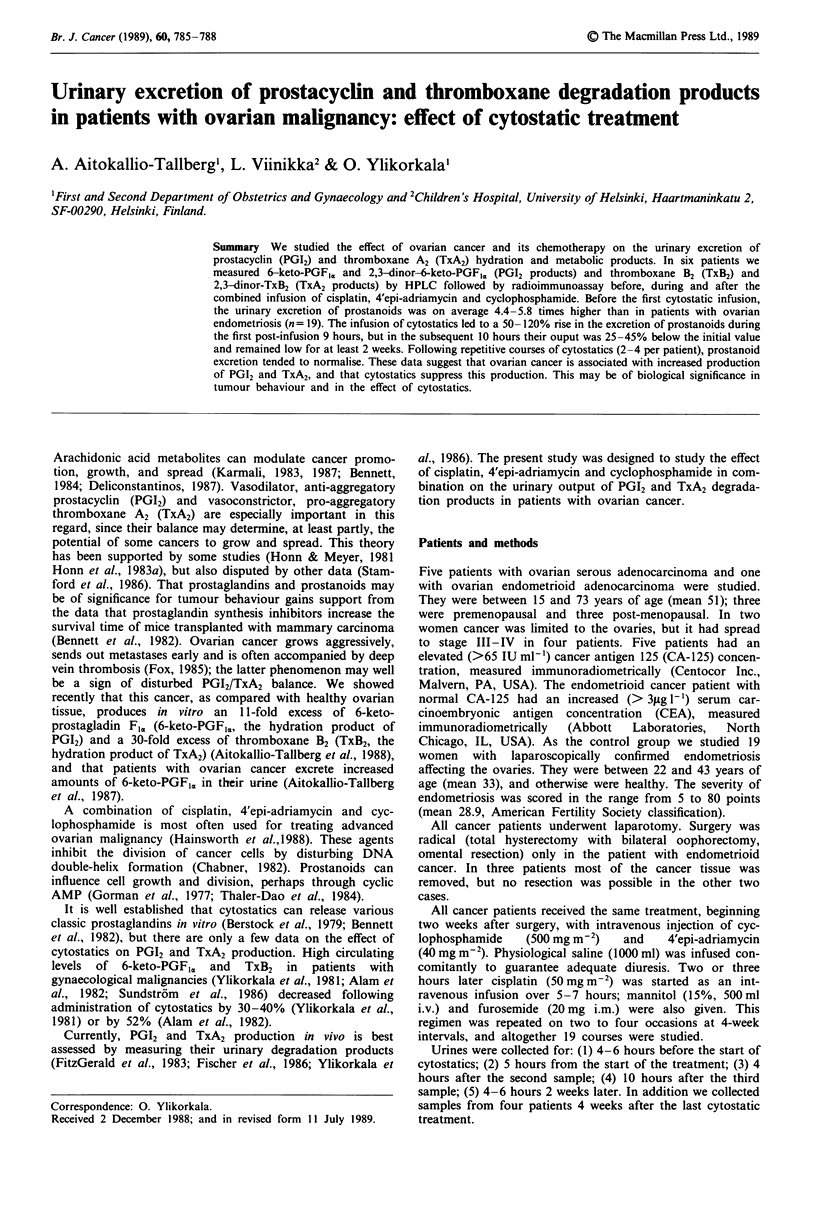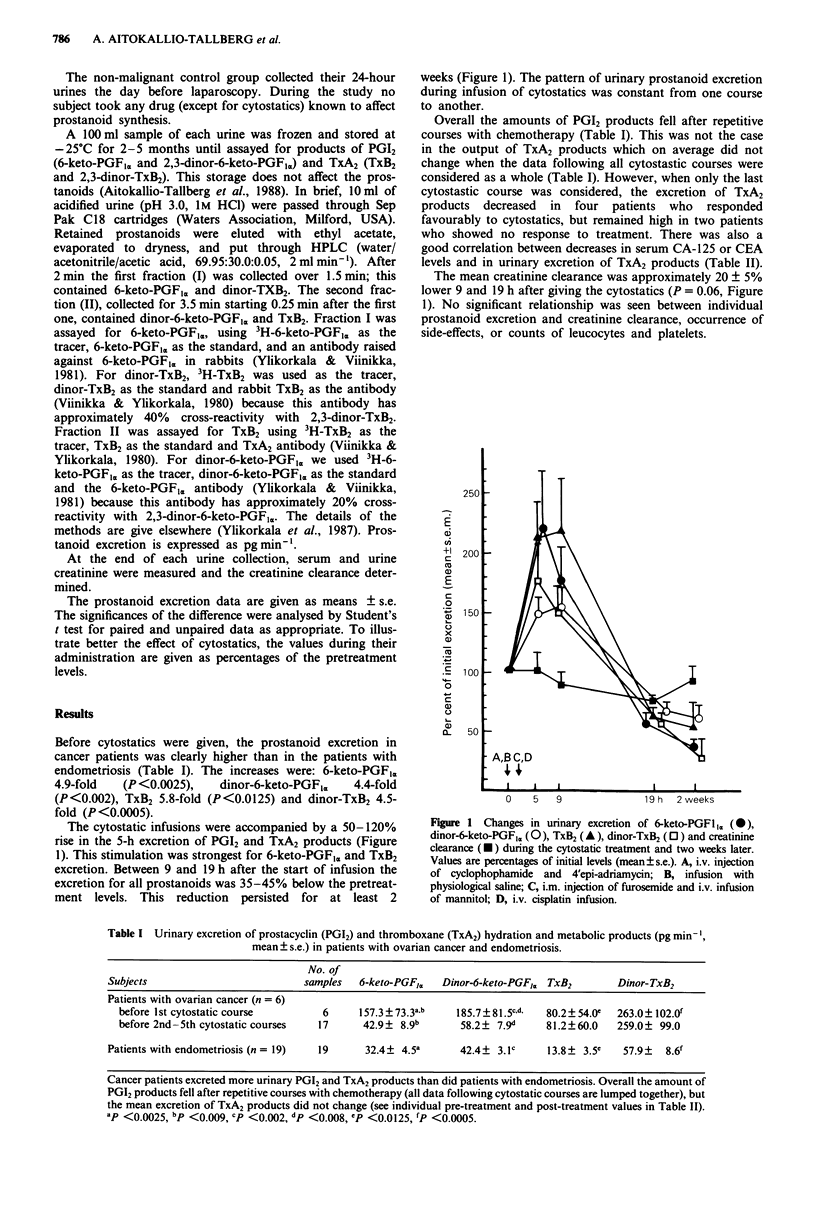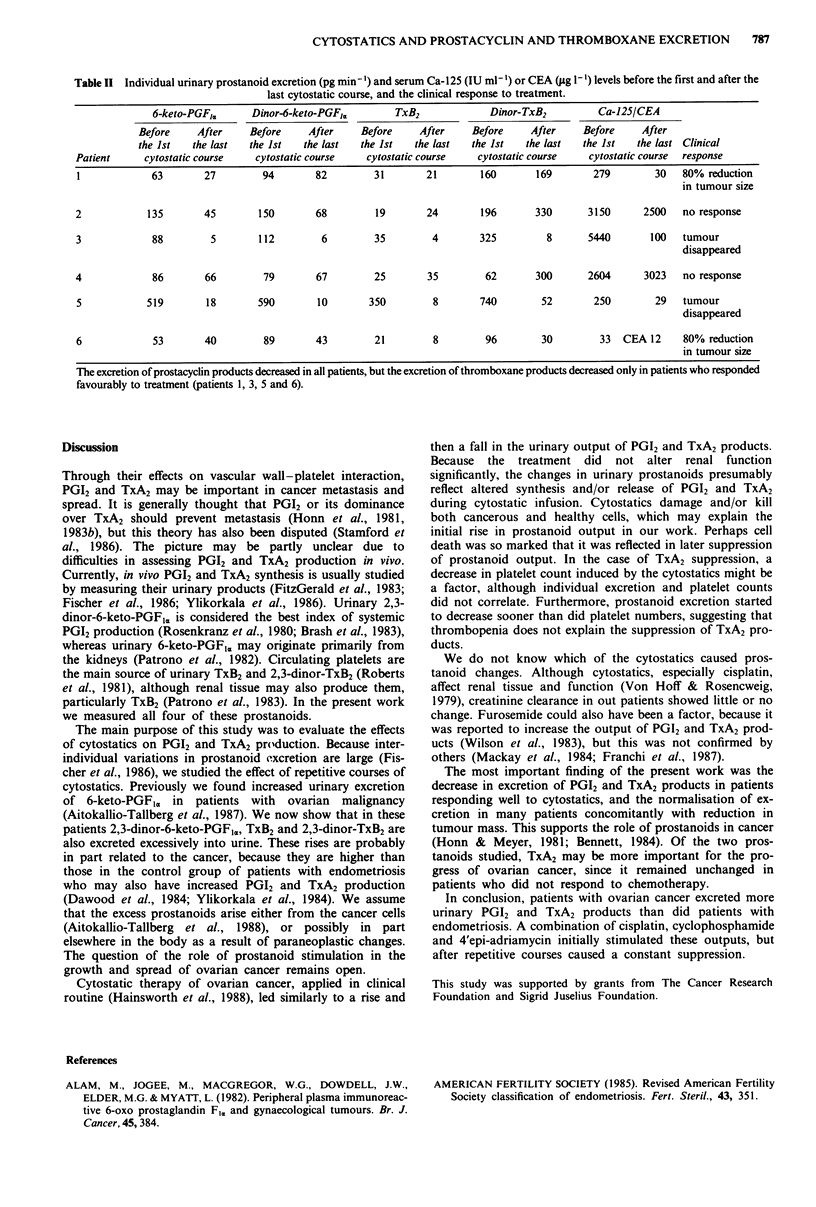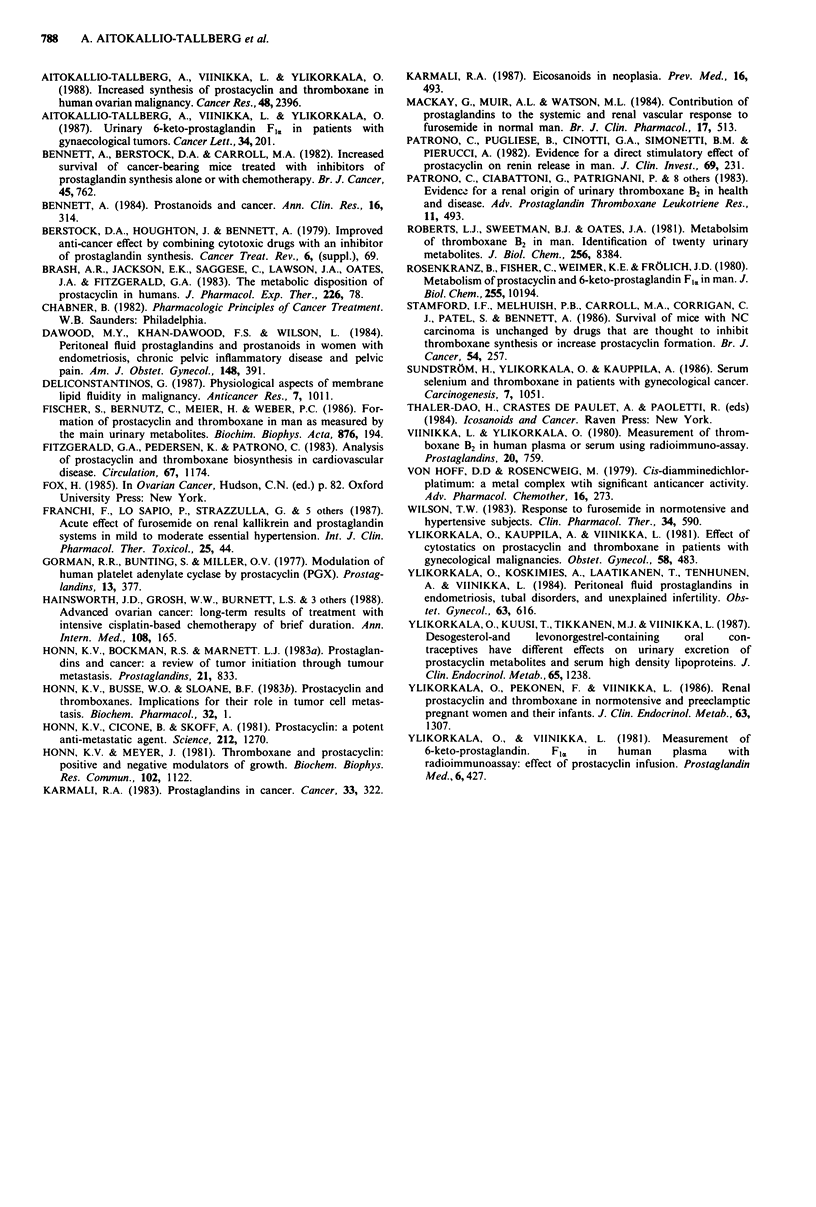# Urinary excretion of prostacyclin and thromboxane degradation products in patients with ovarian malignancy: effect of cytostatic treatment.

**DOI:** 10.1038/bjc.1989.360

**Published:** 1989-11

**Authors:** A. Aitokallio-Tallberg, L. Viinikka, O. Ylikorkala

**Affiliations:** First and Second Department of Obstetrics and Gynaecology, University of Helsinki, Finland.

## Abstract

We studied the effect of ovarian cancer and its chemotherapy on the urinary excretion of prostacyclin (PGI2) and thromboxane A2 (TxA2) hydration and metabolic products. In six patients we measured 6-keto-PGF1 alpha and 2,3-dinor-6-keto-PGF1 alpha (PGI2 products) and thromboxane B2 (TxB2) and 2,3-dinor-TxB2 (TxA2 products) by HPLC followed by radioimmunoassay before, during and after the combined infusion of cisplatin, 4'epi-adriamycin and cyclophosphamide. Before the first cytostatic infusion, the urinary excretion of prostanoids was on average 4.4-5.8 times higher than in patients with ovarian endometriosis (n = 19). The infusion of cytostatics led to a 50-120% rise in the excretion of prostanoids during the first post-infusion 9 hours, but in the subsequent 10 hours their output was 25-45% below the initial value and remained low for at least 2 weeks. Following repetitive courses of cytostatics (2-4 per patient), prostanoid excretion tended to normalise. These data suggest that ovarian cancer is associated with increased production of PGI2 and TxA2, and that cytostatics suppress this production. This may be of biological significance in tumour behaviour and in the effect of cytostatics.


Br. J. Cancer (1989), 60, 785-788 © The Macmillan Press Ltd., 1989

### Urinary excretion of prostacyclin and thromboxane degradation products in patients with ovarian malignancy: effect of cytostatic treatment

A. Aitokallio-Tallberg[1], L. Viinikka[2] & O. Ylikorkala[1]

[1]*First and Second Department of Obstetrics and Gynaecology and* [2]*Children's Hospital, University of Helsinki, Haartmaninkatu 2, SF-00290, Helsinki, Finland.*

**Summary** We studied the effect of ovarian cancer and its chemotherapy on the urinary excretion of prostacyclin ($PGI_2$) and thromboxane $A_2$ ($TxA_2$) hydration and metabolic products. In six patients we measured 6-keto-$PGF_{1\alpha}$ and 2,3-dinor-6-keto-$PGF_{1\alpha}$ ($PGI_2$ products) and thromboxane $B_2$ ($TxB_2$) and 2,3-dinor-$TxB_2$ ($TxA_2$ products) by HPLC followed by radioimmunoassay before, during and after the combined infusion of cisplatin, 4'epi-adriamycin and cyclophosphamide. Before the first cytostatic infusion, the urinary excretion of prostanoids was on average 4.4-5.8 times higher than in patients with ovarian endometriosis ($n = 19$). The infusion of cytostatics led to a 50-120% rise in the excretion of prostanoids during the first post-infusion 9 hours, but in the subsequent 10 hours their ouput was 25-45% below the initial value and remained low for at least 2 weeks. Following repetitive courses of cytostatics (2-4 per patient), prostanoid excretion tended to normalise. These data suggest that ovarian cancer is associated with increased production of $PGI_2$ and $TxA_2$, and that cytostatics suppress this production. This may be of biological significance in tumour behaviour and in the effect of cytostatics.

Arachidonic acid metabolites can modulate cancer promotion, growth, and spread (Karmali, 1983, 1987; Bennett, 1984; Deliconstantinos, 1987). Vasodilator, anti-aggregatory prostacyclin ($PGI_2$) and vasoconstrictor, pro-aggregatory thromboxane $A_2$ ($TxA_2$) are especially important in this regard, since their balance may determine, at least partly, the potential of some cancers to grow and spread. This theory has been supported by some studies (Honn & Meyer, 1981 Honn *et al.*, 1983*a*), but also disputed by other data (Stamford *et al.*, 1986). That prostaglandins and prostanoids may be of significance for tumour behaviour gains support from the data that prostaglandin synthesis inhibitors increase the survival time of mice transplanted with mammary carcinoma (Bennett *et al.*, 1982). Ovarian cancer grows aggressively, sends out metastases early and is often accompanied by deep vein thrombosis (Fox, 1985); the latter phenomenon may well be a sign of disturbed $PGI_2/TxA_2$ balance. We showed recently that this cancer, as compared with healthy ovarian tissue, produces *in vitro* an 11-fold excess of 6-keto-prostagladin $F_{1\alpha}$ (6-keto-$PGF_{1\alpha}$, the hydration product of $PGI_2$) and a 30-fold excess of thromboxane $B_2$ ($TxB_2$, the hydration product of $TxA_2$) (Aitokallio-Tallberg *et al.*, 1988), and that patients with ovarian cancer excrete increased amounts of 6-keto-$PGF_{1\alpha}$ in their urine (Aitokallio-Tallberg *et al.*, 1987).

A combination of cisplatin, 4'epi-adriamycin and cyclophosphamide is most often used for treating advanced ovarian malignancy (Hainsworth *et al.*,1988). These agents inhibit the division of cancer cells by disturbing DNA double-helix formation (Chabner, 1982). Prostanoids can influence cell growth and division, perhaps through cyclic AMP (Gorman *et al.*, 1977; Thaler-Dao *et al.*, 1984).

It is well established that cytostatics can release various classic prostaglandins *in vitro* (Berstock *et al.*, 1979; Bennett *et al.*, 1982), but there are only a few data on the effect of cytostatics on $PGI_2$ and $TxA_2$ production. High circulating levels of 6-keto-$PGF_{1\alpha}$ and $TxB_2$ in patients with gynaecological malignancies (Ylikorkala *et al.*, 1981; Alam *et al.*, 1982; Sundström *et al.*, 1986) decreased following administration of cytostatics by 30-40% (Ylikorkala *et al.*, 1981) or by 52% (Alam *et al.*, 1982).

Currently, $PGI_2$ and $TxA_2$ production *in vivo* is best assessed by measuring their urinary degradation products (FitzGerald *et al.*, 1983; Fischer *et al.*, 1986; Ylikorkala *et al.*, 1986). The present study was designed to study the effect of cisplatin, 4'epi-adriamycin and cyclophosphamide in combination on the urinary output of $PGI_2$ and $TxA_2$ degradation products in patients with ovarian cancer.

### Patients and methods

Five patients with ovarian serous adenocarcinoma and one with ovarian endometrioid adenocarcinoma were studied. They were between 15 and 73 years of age (mean 51); three were premenopausal and three post-menopausal. In two women cancer was limited to the ovaries, but it had spread to stage III-IV in four patients. Five patients had an elevated (>65 IU $ml^{-1}$) cancer antigen 125 (CA-125) concentration, measured immunoradiometrically (Centocor Inc., Malvern, PA, USA). The endometrioid cancer patient with normal CA-125 had an increased (> $3 \mu g\, l^{-1}$) serum carcinoembryonic antigen concentration (CEA), measured immunoradiometrically (Abbott Laboratories, North Chicago, IL, USA). As the control group we studied 19 women with laparoscopically confirmed endometriosis affecting the ovaries. They were between 22 and 43 years of age (mean 33), and otherwise were healthy. The severity of endometriosis was scored in the range from 5 to 80 points (mean 28.9, American Fertility Society classification).

All cancer patients underwent laparotomy. Surgery was radical (total hysterectomy with bilateral oophorectomy, omental resection) only in the patient with endometrioid cancer. In three patients most of the cancer tissue was removed, but no resection was possible in the other two cases.

All cancer patients received the same treatment, beginning two weeks after surgery, with intravenous injection of cyclophosphamide ($500\, mg\, m^{-2}$) and 4'epi-adriamycin ($40\, mg\, m^{-2}$). Physiological saline (1000 ml) was infused concomitantly to guarantee adequate diuresis. Two or three hours later cisplatin ($50\, mg\, m^{-2}$) was started as an intravenous infusion over 5-7 hours; mannitol (15%, 500 ml i.v.) and furosemide (20 mg i.m.) were also given. This regimen was repeated on two to four occasions at 4-week intervals, and altogether 19 courses were studied.

Urines were collected for: (1) 4-6 hours before the start of cytostatics; (2) 5 hours from the start of the treatment; (3) 4 hours after the second sample; (4) 10 hours after the third sample; (5) 4-6 hours 2 weeks later. In addition we collected samples from four patients 4 weeks after the last cytostatic treatment.

Correspondence: O. Ylikorkala.
Received 2 December 1988; and in revised form 11 July 1989.

The non-malignant control group collected their 24-hour urines the day before laparoscopy. During the study no subject took any drug (except for cytostatics) known to affect prostanoid synthesis.

A 100 ml sample of each urine was frozen and stored at −25°C for 2–5 months until assayed for products of $PGI_2$ (6-keto-$PGF_{1\alpha}$ and 2,3-dinor-6-keto-$PGF_{1\alpha}$) and $TxA_2$ ($TxB_2$ and 2,3-dinor-$TxB_2$). This storage does not affect the prostanoids (Aitokallio-Tallberg *et al.*, 1988). In brief, 10 ml of acidified urine (pH 3.0, 1 M HCl) were passed through Sep Pak C18 cartridges (Waters Association, Milford, USA). Retained prostanoids were eluted with ethyl acetate, evaporated to dryness, and put through HPLC (water/acetonitrile/acetic acid, 69.95:30.0:0.05, 2 ml $min^{-1}$). After 2 min the first fraction (I) was collected over 1.5 min; this contained 6-keto-$PGF_{1\alpha}$ and dinor-$TXB_2$. The second fraction (II), collected for 3.5 min starting 0.25 min after the first one, contained dinor-6-keto-$PGF_{1\alpha}$ and $TxB_2$. Fraction I was assayed for 6-keto-$PGF_{1\alpha}$, using $^3H$-6-keto-$PGF_{1\alpha}$ as the tracer, 6-keto-$PGF_{1\alpha}$ as the standard, and an antibody raised against 6-keto-$PGF_{1\alpha}$ in rabbits (Ylikorkala & Viinikka, 1981). For dinor-$TxB_2$, $^3H$-$TxB_2$ was used as the tracer, dinor-$TxB_2$ as the standard and rabbit $TxB_2$ as the antibody (Viinikka & Ylikorkala, 1980) because this antibody has approximately 40% cross-reactivity with 2,3-dinor-$TxB_2$. Fraction II was assayed for $TxB_2$ using $^3H$-$TxB_2$ as the tracer, $TxB_2$ as the standard and $TxA_2$ antibody (Viinikka & Ylikorkala, 1980). For dinor-6-keto-$PGF_{1\alpha}$ we used $^3H$-6-keto-$PGF_{1\alpha}$ as the tracer, dinor-6-keto-$PGF_{1\alpha}$ as the standard and the 6-keto-$PGF_{1\alpha}$ antibody (Ylikorkala & Viinikka, 1981) because this antibody has approximately 20% cross-reactivity with 2,3-dinor-6-keto-$PGF_{1\alpha}$. The details of the methods are give elsewhere (Ylikorkala *et al.*, 1987). Prostanoid excretion is expressed as pg $min^{-1}$.

At the end of each urine collection, serum and urine creatinine were measured and the creatinine clearance determined.

The prostanoid excretion data are given as means ± s.e. The significances of the difference were analysed by Student's *t* test for paired and unpaired data as appropriate. To illustrate better the effect of cytostatics, the values during their administration are given as percentages of the pretreatment levels.

### Results

Before cytostatics were given, the prostanoid excretion in cancer patients was clearly higher than in the patients with endometriosis (Table I). The increases were: 6-keto-$PGF_{1\alpha}$ 4.9-fold ($P<0.0025$), dinor-6-keto-$PGF_{1\alpha}$ 4.4-fold ($P<0.002$), $TxB_2$ 5.8-fold ($P<0.0125$) and dinor-$TxB_2$ 4.5-fold ($P<0.0005$).

The cytostatic infusions were accompanied by a 50–120% rise in the 5-h excretion of $PGI_2$ and $TxA_2$ products (Figure 1). This stimulation was strongest for 6-keto-$PGF_{1\alpha}$ and $TxB_2$ excretion. Between 9 and 19 h after the start of infusion the excretion for all prostanoids was 35–45% below the pretreatment levels. This reduction persisted for at least 2 weeks (Figure 1). The pattern of urinary prostanoid excretion during infusion of cytostatics was constant from one course to another.

Overall the amounts of $PGI_2$ products fell after repetitive courses with chemotherapy (Table I). This was not the case in the output of $TxA_2$ products which on average did not change when the data following all cytostastic courses were considered as a whole (Table I). However, when only the last cytostastic course was considered, the excretion of $TxA_2$ products decreased in four patients who responded favourably to cytostatics, but remained high in two patients who showed no response to treatment. There was also a good correlation between decreases in serum CA-125 or CEA levels and in urinary excretion of $TxA_2$ products (Table II).

The mean creatinine clearance was approximately 20 ± 5% lower 9 and 19 h after giving the cytostatics ($P = 0.06$, Figure 1). No significant relationship was seen between individual prostanoid excretion and creatinine clearance, occurrence of side-effects, or counts of leucocytes and platelets.

**Figure 1** Changes in urinary excretion of 6-keto-$PGF1_{1\alpha}$ (●), dinor-6-keto-$PGF_{1\alpha}$ (○), $TxB_2$ (▲), dinor-$TxB_2$ (□) and creatinine clearance (■) during the cytostatic treatment and two weeks later. Values are percentages of initial levels (mean±s.e.). **A**, i.v. injection of cyclophophamide and 4'epi-adriamycin; **B**, infusion with physiological saline; **C**, i.m. injection of furosemide and i.v. infusion of mannitol; **D**, i.v. cisplatin infusion.

**Table I** Urinary excretion of prostacyclin ($PGI_2$) and thromboxane ($TxA_2$) hydration and metabolic products (pg $min^{-1}$, mean ± s.e.) in patients with ovarian cancer and endometriosis.

| *Subjects* | *No. of samples* | *6-keto-$PGF_{1\alpha}$* | *Dinor-6-keto-$PGF_{1\alpha}$* | *$TxB_2$* | *Dinor-$TxB_2$* |
|---|---|---|---|---|---|
| Patients with ovarian cancer ($n = 6$) | | | | | |
| before 1st cytostatic course | 6 | 157.3±73.3[a,b] | 185.7±81.5[c,d] | 80.2±54.0[e] | 263.0±102.0[f] |
| before 2nd–5th cytostatic courses | 17 | 42.9± 8.9[b] | 58.2± 7.9[d] | 81.2±60.0 | 259.0± 99.0 |
| Patients with endometriosis ($n = 19$) | 19 | 32.4± 4.5[a] | 42.4± 3.1[c] | 13.8± 3.5[e] | 57.9± 8.6[f] |

Cancer patients excreted more urinary $PGI_2$ and $TxA_2$ products than did patients with endometriosis. Overall the amount of $PGI_2$ products fell after repetitive courses with chemotherapy (all data following cytostatic courses are lumped together), but the mean excretion of $TxA_2$ products did not change (see individual pre-treatment and post-treatment values in Table II).
[a]$P<0.0025$, [b]$P<0.009$, [c]$P<0.002$, [d]$P<0.008$, [e]$P<0.0125$, [f]$P<0.0005$.

**Table II** Individual urinary prostanoid excretion (pg $min^{-1}$) and serum Ca-125 (IU $ml^{-1}$) or CEA ($\mu g\ l^{-1}$) levels before the first and after the last cytostatic course, and the clinical response to treatment.

| | *6-keto-PGF$_{1\alpha}$* | | *Dinor-6-keto-PGF$_{1\alpha}$* | | *TxB$_2$* | | *Dinor-TxB$_2$* | | *Ca-125/CEA* | | |
|---|---|---|---|---|---|---|---|---|---|---|---|
| *Patient* | *Before the 1st cytostatic course* | *After the last cytostatic course* | *Before the 1st cytostatic course* | *After the last cytostatic course* | *Before the 1st cytostatic course* | *After the last cytostatic course* | *Before the 1st cytostatic course* | *After the last cytostatic course* | *Before the 1st cytostatic course* | *After the last cytostatic course* | *Clinical response* |
| 1 | 63 | 27 | 94 | 82 | 31 | 21 | 160 | 169 | 279 | 30 | 80% reduction in tumour size |
| 2 | 135 | 45 | 150 | 68 | 19 | 24 | 196 | 330 | 3150 | 2500 | no response |
| 3 | 88 | 5 | 112 | 6 | 35 | 4 | 325 | 8 | 5440 | 100 | tumour disappeared |
| 4 | 86 | 66 | 79 | 67 | 25 | 35 | 62 | 300 | 2604 | 3023 | no response |
| 5 | 519 | 18 | 590 | 10 | 350 | 8 | 740 | 52 | 250 | 29 | tumour disappeared |
| 6 | 53 | 40 | 89 | 43 | 21 | 8 | 96 | 30 | 33 | CEA 12 | 80% reduction in tumour size |

The excretion of prostacyclin products decreased in all patients, but the excretion of thromboxane products decreased only in patients who responded favourably to treatment (patients 1, 3, 5 and 6).

### Discussion

Through their effects on vascular wall–platelet interaction, $PGI_2$ and $TxA_2$ may be important in cancer metastasis and spread. It is generally thought that $PGI_2$ or its dominance over $TxA_2$ should prevent metastasis (Honn *et al.*, 1981, 1983*b*), but this theory has also been disputed (Stamford *et al.*, 1986). The picture may be partly unclear due to difficulties in assessing $PGI_2$ and $TxA_2$ production *in vivo*. Currently, *in vivo* $PGI_2$ and $TxA_2$ synthesis is usually studied by measuring their urinary products (FitzGerald *et al.*, 1983; Fischer *et al.*, 1986; Ylikorkala *et al.*, 1986). Urinary 2,3-dinor-6-keto-$PGF_{1\alpha}$ is considered the best index of systemic $PGI_2$ production (Rosenkranz *et al.*, 1980; Brash *et al.*, 1983), whereas urinary 6-keto-$PGF_{1\alpha}$ may originate primarily from the kidneys (Patrono *et al.*, 1982). Circulating platelets are the main source of urinary $TxB_2$ and 2,3-dinor-$TxB_2$ (Roberts *et al.*, 1981), although renal tissue may also produce them, particularly $TxB_2$ (Patrono *et al.*, 1983). In the present work we measured all four of these prostanoids.

The main purpose of this study was to evaluate the effects of cytostatics on $PGI_2$ and $TxA_2$ production. Because inter-individual variations in prostanoid excretion are large (Fischer *et al.*, 1986), we studied the effect of repetitive courses of cytostatics. Previously we found increased urinary excretion of 6-keto-$PGF_{1\alpha}$ in patients with ovarian malignancy (Aitokallio-Tallberg *et al.*, 1987). We now show that in these patients 2,3-dinor-6-keto-$PGF_{1\alpha}$, $TxB_2$ and 2,3-dinor-$TxB_2$ are also excreted excessively into urine. These rises are probably in part related to the cancer, because they are higher than those in the control group of patients with endometriosis who may also have increased $PGI_2$ and $TxA_2$ production (Dawood *et al.*, 1984; Ylikorkala *et al.*, 1984). We assume that the excess prostanoids arise either from the cancer cells (Aitokallio-Tallberg *et al.*, 1988), or possibly in part elsewhere in the body as a result of paraneoplastic changes. The question of the role of prostanoid stimulation in the growth and spread of ovarian cancer remains open.

Cytostatic therapy of ovarian cancer, applied in clinical routine (Hainsworth *et al.*, 1988), led similarly to a rise and then a fall in the urinary output of $PGI_2$ and $TxA_2$ products. Because the treatment did not alter renal function significantly, the changes in urinary prostanoids presumably reflect altered synthesis and/or release of $PGI_2$ and $TxA_2$ during cytostatic infusion. Cytostatics damage and/or kill both cancerous and healthy cells, which may explain the initial rise in prostanoid output in our work. Perhaps cell death was so marked that it was reflected in later suppression of prostanoid output. In the case of $TxA_2$ suppression, a decrease in platelet count induced by the cytostatics might be a factor, although individual excretion and platelet counts did not correlate. Furthermore, prostanoid excretion started to decrease sooner than did platelet numbers, suggesting that thrombopenia does not explain the suppression of $TxA_2$ products.

We do not know which of the cytostatics caused prostanoid changes. Although cytostatics, especially cisplatin, affect renal tissue and function (Von Hoff & Rosencweig, 1979), creatinine clearance in out patients showed little or no change. Furosemide could also have been a factor, because it was reported to increase the output of $PGI_2$ and $TxA_2$ products (Wilson *et al.*, 1983), but this was not confirmed by others (Mackay *et al.*, 1984; Franchi *et al.*, 1987).

The most important finding of the present work was the decrease in excretion of $PGI_2$ and $TxA_2$ products in patients responding well to cytostatics, and the normalisation of excretion in many patients concomitantly with reduction in tumour mass. This supports the role of prostanoids in cancer (Honn & Meyer, 1981; Bennett, 1984). Of the two prostanoids studied, $TxA_2$ may be more important for the progress of ovarian cancer, since it remained unchanged in patients who did not respond to chemotherapy.

In conclusion, patients with ovarian cancer excreted more urinary $PGI_2$ and $TxA_2$ products than did patients with endometriosis. A combination of cisplatin, cyclophosphamide and 4'epi-adriamycin initially stimulated these outputs, but after repetitive courses caused a constant suppression.

This study was supported by grants from The Cancer Research Foundation and Sigrid Juselius Foundation.

### References

ALAM, M., JOGEE, M., MACGREGOR, W.G., DOWDELL, J.W., ELDER, M.G. & MYATT, L. (1982). Peripheral plasma immunoreactive 6-oxo prostaglandin $F_{1\alpha}$ and gynaecological tumours. *Br. J. Cancer*, **45**, 384.

AMERICAN FERTILITY SOCIETY (1985). Revised American Fertility Society classification of endometriosis. *Fert. Steril.*, **43**, 351.

AITOKALLIO-TALLBERG, A., VIINIKKA, L. & YLIKORKALA, O. (1988). Increased synthesis of prostacyclin and thromboxane in human ovarian malignancy. *Cancer Res.*, **48**, 2396.

AITOKALLIO-TALLBERG, A., VIINIKKA, L. & YLIKORKALA, O. (1987). Urinary 6-keto-prostaglandin $F_{1\alpha}$ in patients with gynaecological tumors. *Cancer Lett.*, **34**, 201.

BENNETT, A., BERSTOCK, D.A. & CARROLL, M.A. (1982). Increased survival of cancer-bearing mice treated with inhibitors of prostaglandin synthesis alone or with chemotherapy. *Br. J. Cancer*, **45**, 762.

BENNETT, A. (1984). Prostanoids and cancer. *Ann. Clin. Res.*, **16**, 314.

BERSTOCK, D.A., HOUGHTON, J. & BENNETT, A. (1979). Improved anti-cancer effect by combining cytotoxic drugs with an inhibitor of prostaglandin synthesis. *Cancer Treat. Rev.*, **6**, (suppl.), 69.

BRASH, A.R., JACKSON, E.K., SAGGESE, C., LAWSON, J.A., OATES, J.A. & FITZGERALD, G.A. (1983). The metabolic disposition of prostacyclin in humans. *J. Pharmacol. Exp. Ther.*, **226**, 78.

CHABNER, B. (1982). *Pharmacologic Principles of Cancer Treatment.* W.B. Saunders: Philadelphia.

DAWOOD, M.Y., KHAN-DAWOOD, F.S. & WILSON, L. (1984). Peritoneal fluid prostaglandins and prostanoids in women with endometriosis, chronic pelvic inflammatory disease and pelvic pain. *Am. J. Obstet. Gynecol.*, **148**, 391.

DELICONSTANTINOS, G. (1987). Physiological aspects of membrane lipid fluidity in malignancy. *Anticancer Res.*, **7**, 1011.

FISCHER, S., BERNUTZ, C., MEIER, H. & WEBER, P.C. (1986). Formation of prostacyclin and thromboxane in man as measured by the main urinary metabolites. *Biochim. Biophys. Acta*, **876**, 194.

FITZGERALD, G.A., PEDERSEN, K. & PATRONO, C. (1983). Analysis of prostacyclin and thromboxane biosynthesis in cardiovascular disease. *Circulation*, **67**, 1174.

FOX, H. (1985). In *Ovarian Cancer*, Hudson, C.N. (ed.) p. 82. Oxford University Press: New York.

FRANCHI, F., LO SAPIO, P., STRAZZULLA, G. & 5 others (1987). Acute effect of furosemide on renal kallikrein and prostaglandin systems in mild to moderate essential hypertension. *Int. J. Clin. Pharmacol. Ther. Toxicol.*, **25**, 44.

GORMAN, R.R., BUNTING, S. & MILLER, O.V. (1977). Modulation of human platelet adenylate cyclase by prostacyclin (PGX). *Prostaglandins*, **13**, 377.

HAINSWORTH, J.D., GROSH, W.W., BURNETT, L.S. & 3 others (1988). Advanced ovarian cancer: long-term results of treatment with intensive cisplatin-based chemotherapy of brief duration. *Ann. Intern. Med.*, **108**, 165.

HONN, K.V., BOCKMAN, R.S. & MARNETT. L.J. (1983*a*). Prostaglandins and cancer: a review of tumor initiation through tumour metastasis. *Prostaglandins*, **21**, 833.

HONN, K.V., BUSSE, W.O. & SLOANE, B.F. (1983*b*). Prostacyclin and thromboxanes. Implications for their role in tumor cell metastasis. *Biochem. Pharmacol.*, **32**, 1.

HONN, K.V., CICONE, B. & SKOFF, A. (1981). Prostacyclin: a potent anti-metastatic agent. *Science*, **212**, 1270.

HONN, K.V. & MEYER, J. (1981). Thromboxane and prostacyclin: positive and negative modulators of growth. *Biochem. Biophys. Res. Commun.*, **102**, 1122.

KARMALI, R.A. (1983). Prostaglandins in cancer. *Cancer*, **33**, 322.

KARMALI, R.A. (1987). Eicosanoids in neoplasia. *Prev. Med.*, **16**, 493.

MACKAY, G., MUIR, A.L. & WATSON, M.L. (1984). Contribution of prostaglandins to the systemic and renal vascular response to furosemide in normal man. *Br. J. Clin. Pharmacol.*, **17**, 513.

PATRONO, C., PUGLIESE, B., CINOTTI, G.A., SIMONETTI, B.M. & PIERUCCI, A. (1982). Evidence for a direct stimulatory effect of prostacyclin on renin release in man. *J. Clin. Invest.*, **69**, 231.

PATRONO, C., CIABATTONI, G., PATRIGNANI, P. & 8 others (1983). Evidence for a renal origin of urinary thromboxane $B_2$ in health and disease. *Adv. Prostaglandin Thromboxane Leukotriene Res.*, **11**, 493.

ROBERTS, L.J., SWEETMAN, B.J. & OATES, J.A. (1981). Metabolsim of thromboxane $B_2$ in man. Identification of twenty urinary metabolites. *J. Biol. Chem.*, **256**, 8384.

ROSENKRANZ, B., FISHER, C., WEIMER, K.E. & FRÖLICH, J.D. (1980). Metabolism of prostacyclin and 6-keto-prostaglandin $F_{1\alpha}$ in man. *J. Biol. Chem.*, **255**, 10194.

STAMFORD, I.F., MELHUISH, P.B., CARROLL, M.A., CORRIGAN, C. J., PATEL, S. & BENNETT, A. (1986). Survival of mice with NC carcinoma is unchanged by drugs that are thought to inhibit thromboxane synthesis or increase prostacyclin formation. *Br. J. Cancer*, **54**, 257.

SUNDSTRÖM, H., YLIKORKALA, O. & KAUPPILA, A. (1986). Serum selenium and thromboxane in patients with gynecological cancer. *Carcinogenesis*, **7**, 1051.

THALER-DAO, H., CRASTES DE PAULET, A. & PAOLETTI, R. (eds) (1984). *Icosanoids and Cancer.* Raven Press: New York.

VIINIKKA, L. & YLIKORKALA, O. (1980). Measurement of thromboxane $B_2$ in human plasma or serum using radioimmuno-assay. *Prostaglandins*, **20**, 759.

VON HOFF, D.D & ROSENCWEIG, M. (1979). *Cis*-diamminedichlorplatimum: a metal complex wtih significant anticancer activity. *Adv. Pharmacol. Chemother*, **16**, 273.

WILSON, T.W. (1983). Response to furosemide in normotensive and hypertensive subjects. *Clin. Pharmacol. Ther.*, **34**, 590.

YLIKORKALA, O., KAUPPILA, A. & VIINIKKA, L. (1981). Effect of cytostatics on prostacyclin and thromboxane in patients with gynecological malignancies. *Obstet. Gynecol.*, **58**, 483.

YLIKORKALA, O., KOSKIMIES, A., LAATIKANEN, T., TENHUNEN, A. & VIINIKKA, L. (1984). Peritoneal fluid prostaglandins in endometriosis, tubal disorders, and unexplained infertility. *Obstet. Gynecol.*, **63**, 616.

YLIKORKALA, O., KUUSI, T., TIKKANEN, M.J. & VIINIKKA, L. (1987). Desogesterol-and levonorgestrel-containing oral contraceptives have different effects on urinary excretion of prostacyclin metabolites and serum high density lipoproteins. *J. Clin. Endocrinol. Metab.*, **65**, 1238.

YLIKORKALA, O., PEKONEN, F. & VIINIKKA, L. (1986). Renal prostacyclin and thromboxane in normotensive and preeclamptic pregnant women and their infants. *J. Clin. Endocrinol. Metab.*, **63**, 1307.

YLIKORKALA, O., & VIINIKKA, L. (1981). Measurement of 6-keto-prostaglandin. $F_{1\alpha}$ in human plasma with radioimmunoassay: effect of prostacyclin infusion. *Prostaglandin Med.*, **6**, 427.